# Supplementary material for: Modeling glioblastoma heterogeneity as a dynamic network of cell states
Source: Mol Syst Biol. 2021 Sep 16;17(9):e10105. doi: 10.15252/msb.202010105 (PMC8444284; doi:10.15252/msb.202010105)
Supplement: Supplementary file 6 — Source Data for Figure 5 [file MSB-17-e10105-s004.zip › Figure5A_sourcedata/GSEA_3071/nosigdb_stateV.GseaPreranked.1622795159954/RICHARDS_ZHONG_NATURE_2018_NPCS_UPREG.html]

Details for gene set RICHARDS\_ZHONG\_NATURE\_2018\_NPCS\_UPREG[GSEA]

|  || Dataset | state43071 |
| Phenotype | NoPhenotypeAvailable |
| Upregulated in class | na\_pos |
| GeneSet | RICHARDS\_ZHONG\_NATURE\_2018\_NPCS\_UPREG |
| Enrichment Score (ES) | 0.7173247 |
| Normalized Enrichment Score (NES) | 4.0820146 |
| Nominal p-value | 0.0 |
| FDR q-value | 0.0 |
| FWER p-Value | 0.0 |
Table: GSEA Results Summary

  

Fig 1: Enrichment plot: RICHARDS\_ZHONG\_NATURE\_2018\_NPCS\_UPREG      
 Profile of the Running ES Score & Positions of GeneSet Members on the Rank Ordered List

  

| PROBE | GENE SYMBOL | GENE\_TITLE | RANK IN GENE LIST | RANK METRIC SCORE | RUNNING ES | CORE ENRICHMENT || 1 | HIST1H4C |  |  | 1 | 2.442 | 0.0188 | Yes |
| 2 | ATAD2 |  |  | 2 | 2.214 | 0.0361 | Yes |
| 3 | HMGB2 |  |  | 5 | 2.011 | 0.0514 | Yes |
| 4 | NUSAP1 |  |  | 6 | 2.001 | 0.0670 | Yes |
| 5 | PCNA |  |  | 10 | 1.908 | 0.0813 | Yes |
| 6 | TOP2A |  |  | 14 | 1.842 | 0.0951 | Yes |
| 7 | MCM4 |  |  | 17 | 1.815 | 0.1089 | Yes |
| 8 | DHFR |  |  | 18 | 1.795 | 0.1229 | Yes |
| 9 | C21orf58 |  |  | 20 | 1.772 | 0.1365 | Yes |
| 10 | H2AFX |  |  | 26 | 1.725 | 0.1490 | Yes |
| 11 | CENPF |  |  | 28 | 1.705 | 0.1621 | Yes |
| 12 | RRM2 |  |  | 34 | 1.660 | 0.1740 | Yes |
| 13 | CENPU |  |  | 36 | 1.645 | 0.1867 | Yes |
| 14 | MKI67 |  |  | 45 | 1.598 | 0.1976 | Yes |
| 15 | HELLS |  |  | 48 | 1.588 | 0.2096 | Yes |
| 16 | MCM3 |  |  | 49 | 1.586 | 0.2219 | Yes |
| 17 | KIF15 |  |  | 52 | 1.568 | 0.2338 | Yes |
| 18 | PBK |  |  | 54 | 1.536 | 0.2456 | Yes |
| 19 | GINS2 |  |  | 57 | 1.528 | 0.2571 | Yes |
| 20 | GTSE1 |  |  | 66 | 1.491 | 0.2672 | Yes |
| 21 | CDK1 |  |  | 69 | 1.488 | 0.2784 | Yes |
| 22 | SMC2 |  |  | 73 | 1.466 | 0.2893 | Yes |
| 23 | SPC25 |  |  | 76 | 1.442 | 0.3001 | Yes |
| 24 | UBE2T |  |  | 79 | 1.432 | 0.3109 | Yes |
| 25 | FBXO5 |  |  | 82 | 1.422 | 0.3216 | Yes |
| 26 | CLSPN |  |  | 84 | 1.417 | 0.3324 | Yes |
| 27 | MCM7 |  |  | 88 | 1.400 | 0.3428 | Yes |
| 28 | RRM1 |  |  | 99 | 1.364 | 0.3515 | Yes |
| 29 | CENPM |  |  | 114 | 1.337 | 0.3592 | Yes |
| 30 | TACC3 |  |  | 116 | 1.334 | 0.3694 | Yes |
| 31 | CDCA5 |  |  | 123 | 1.326 | 0.3786 | Yes |
| 32 | SMC4 |  |  | 127 | 1.320 | 0.3883 | Yes |
| 33 | FEN1 |  |  | 139 | 1.296 | 0.3963 | Yes |
| 34 | GMNN |  |  | 146 | 1.280 | 0.4051 | Yes |
| 35 | MIS18BP1 |  |  | 159 | 1.247 | 0.4125 | Yes |
| 36 | TMPO |  |  | 161 | 1.243 | 0.4220 | Yes |
| 37 | CENPK |  |  | 162 | 1.241 | 0.4317 | Yes |
| 38 | CDKN3 |  |  | 163 | 1.239 | 0.4413 | Yes |
| 39 | KIF23 |  |  | 167 | 1.235 | 0.4504 | Yes |
| 40 | CDCA3 |  |  | 169 | 1.230 | 0.4598 | Yes |
| 41 | NUF2 |  |  | 192 | 1.189 | 0.4648 | Yes |
| 42 | ZWINT |  |  | 204 | 1.166 | 0.4718 | Yes |
| 43 | ASPM |  |  | 207 | 1.163 | 0.4804 | Yes |
| 44 | PRC1 |  |  | 211 | 1.161 | 0.4889 | Yes |
| 45 | KIF22 |  |  | 213 | 1.157 | 0.4977 | Yes |
| 46 | UBE2C |  |  | 214 | 1.154 | 0.5067 | Yes |
| 47 | BTG3 |  |  | 226 | 1.141 | 0.5135 | Yes |
| 48 | MCM2 |  |  | 238 | 1.130 | 0.5202 | Yes |
| 49 | NDC80 |  |  | 240 | 1.129 | 0.5288 | Yes |
| 50 | MCM5 |  |  | 243 | 1.127 | 0.5372 | Yes |
| 51 | TPX2 |  |  | 248 | 1.122 | 0.5452 | Yes |
| 52 | BUB1 |  |  | 277 | 1.084 | 0.5482 | Yes |
| 53 | MCM6 |  |  | 282 | 1.080 | 0.5559 | Yes |
| 54 | CKAP2L |  |  | 285 | 1.078 | 0.5639 | Yes |
| 55 | AURKB |  |  | 297 | 1.068 | 0.5701 | Yes |
| 56 | CCNA2 |  |  | 309 | 1.057 | 0.5762 | Yes |
| 57 | PAX6 |  |  | 311 | 1.055 | 0.5842 | Yes |
| 58 | CKAP2 |  |  | 312 | 1.053 | 0.5925 | Yes |
| 59 | DUT |  |  | 314 | 1.050 | 0.6004 | Yes |
| 60 | PRR11 |  |  | 317 | 1.048 | 0.6082 | Yes |
| 61 | CDCA7 |  |  | 330 | 1.038 | 0.6140 | Yes |
| 62 | ZFHX4 |  |  | 362 | 0.996 | 0.6158 | Yes |
| 63 | KIF11 |  |  | 369 | 0.982 | 0.6223 | Yes |
| 64 | APOLD1 |  |  | 372 | 0.980 | 0.6295 | Yes |
| 65 | DEPDC1B |  |  | 387 | 0.963 | 0.6343 | Yes |
| 66 | UHRF1 |  |  | 390 | 0.958 | 0.6414 | Yes |
| 67 | RPA3 |  |  | 395 | 0.953 | 0.6481 | Yes |
| 68 | HJURP |  |  | 406 | 0.945 | 0.6535 | Yes |
| 69 | DLGAP5 |  |  | 418 | 0.936 | 0.6587 | Yes |
| 70 | MAD2L1 |  |  | 421 | 0.932 | 0.6656 | Yes |
| 71 | KPNA2 |  |  | 435 | 0.919 | 0.6702 | Yes |
| 72 | ORC6 |  |  | 449 | 0.908 | 0.6748 | Yes |
| 73 | KNSTRN |  |  | 457 | 0.902 | 0.6805 | Yes |
| 74 | HMGN2 |  |  | 466 | 0.900 | 0.6859 | Yes |
| 75 | SPAG5 |  |  | 481 | 0.884 | 0.6901 | Yes |
| 76 | DACH1 |  |  | 521 | 0.851 | 0.6892 | Yes |
| 77 | BIRC5 |  |  | 541 | 0.842 | 0.6921 | Yes |
| 78 | ID4 |  |  | 568 | 0.819 | 0.6935 | Yes |
| 79 | CDCA4 |  |  | 615 | 0.790 | 0.6907 | Yes |
| 80 | OIP5 |  |  | 620 | 0.788 | 0.6961 | Yes |
| 81 | WDR34 |  |  | 671 | 0.762 | 0.6924 | Yes |
| 82 | NOTCH1 |  |  | 673 | 0.762 | 0.6981 | Yes |
| 83 | ANP32E |  |  | 722 | 0.739 | 0.6946 | Yes |
| 84 | HAT1 |  |  | 741 | 0.727 | 0.6968 | Yes |
| 85 | DTYMK |  |  | 800 | 0.700 | 0.6911 | Yes |
| 86 | TGIF1 |  |  | 840 | 0.683 | 0.6888 | Yes |
| 87 | H2AFZ |  |  | 851 | 0.677 | 0.6922 | Yes |
| 88 | RNASEH2A |  |  | 859 | 0.674 | 0.6961 | Yes |
| 89 | C19orf48 |  |  | 893 | 0.661 | 0.6949 | Yes |
| 90 | SOX9 |  |  | 905 | 0.658 | 0.6979 | Yes |
| 91 | MELK |  |  | 915 | 0.653 | 0.7012 | Yes |
| 92 | H2AFV |  |  | 933 | 0.647 | 0.7030 | Yes |
| 93 | PTTG1 |  |  | 936 | 0.647 | 0.7076 | Yes |
| 94 | MDK |  |  | 950 | 0.640 | 0.7101 | Yes |
| 95 | CENPN |  |  | 953 | 0.638 | 0.7147 | Yes |
| 96 | CKS2 |  |  | 966 | 0.634 | 0.7173 | Yes |
| 97 | TUBA1B |  |  | 1012 | 0.617 | 0.7134 | No |
| 98 | SOX3 |  |  | 1018 | 0.615 | 0.7173 | No |
| 99 | HMGB3 |  |  | 1154 | 0.577 | 0.6957 | No |
| 100 | ITGB3BP |  |  | 1180 | 0.568 | 0.6953 | No |
| 101 | KIF2C |  |  | 1309 | 0.530 | 0.6747 | No |
| 102 | CCNB2 |  |  | 1373 | 0.513 | 0.6665 | No |
| 103 | CKS1B |  |  | 1502 | 0.479 | 0.6455 | No |
| 104 | GLI3 |  |  | 1569 | 0.466 | 0.6364 | No |
| 105 | TUBB4B |  |  | 1659 | 0.444 | 0.6226 | No |
| 106 | UCP2 |  |  | 1779 | 0.422 | 0.6029 | No |
| 107 | CENPW |  |  | 1959 | 0.395 | 0.5714 | No |
| 108 | CNTLN |  |  | 2004 | 0.387 | 0.5659 | No |
| 109 | CDON |  |  | 2027 | 0.384 | 0.5646 | No |
| 110 | PAICS |  |  | 2403 | 0.334 | 0.4947 | No |
| 111 | CCNB1 |  |  | 2480 | 0.325 | 0.4826 | No |
| 112 | SFRP1 |  |  | 2591 | 0.312 | 0.4637 | No |
| 113 | BUB3 |  |  | 2806 | 0.288 | 0.4246 | No |
| 114 | VIM |  |  | 3830 | -0.354 | 0.2296 | No |
| 115 | RPS27L |  |  | 3927 | -0.373 | 0.2140 | No |
| 116 | TMEM98 |  |  | 4140 | -0.420 | 0.1763 | No |
| 117 | LDHA |  |  | 4145 | -0.421 | 0.1788 | No |
| 118 | RCN1 |  |  | 4276 | -0.467 | 0.1573 | No |
| 119 | TAGLN2 |  |  | 4648 | -0.618 | 0.0904 | No |
| 120 | CLIC1 |  |  | 4899 | -0.809 | 0.0484 | No |
| 121 | HSPB1 |  |  | 4992 | -0.920 | 0.0378 | No |
| 122 | IGF2BP1 |  |  | 5048 | -1.029 | 0.0351 | No |
| 123 | MFAP2 |  |  | 5208 | -1.592 | 0.0168 | No |
Table: GSEA details [plain text format]

  

Fig 2: RICHARDS\_ZHONG\_NATURE\_2018\_NPCS\_UPREG: Random ES distribution      
 Gene set null distribution of ES for **RICHARDS\_ZHONG\_NATURE\_2018\_NPCS\_UPREG**
